# Corpus callosum morphology in major mental disorders: a magnetic resonance imaging study

**DOI:** 10.1093/braincomms/fcab100

**Published:** 2021-05-11

**Authors:** Fabrizio Piras, Daniela Vecchio, Florian Kurth, Federica Piras, Nerisa Banaj, Valentina Ciullo, Eileen Luders, Gianfranco Spalletta

**Affiliations:** 1 Laboratory of Neuropsychiatry, IRCCS Santa Lucia Foundation, 00179 Rome, Italy; 2 School of Psychology, University of Auckland, Auckland, Private Bag 92019, New Zealand; 3 Laboratory of Neuro Imaging, School of Medicine, University of Southern California, Los Angeles, CA, USA; 4 Menninger Department of Psychiatry and Behavioral Sciences, Division of Neuropsychiatry, Baylor College of Medicine, Houston, TX 77030, USA

**Keywords:** corpus callosum, obsessive-compulsive disorder, major depression, schizophrenia, bipolar disorder

## Abstract

Mental disorders diagnosis is based on specific clinical criteria. However, clinical studies found similarities and overlapping phenomenology across a variety of disorders, which suggests a common neurobiological substrate. Thus, there is a need to measure disease-related neuroanatomical similarities and differences across conditions. While structural alterations of the corpus callosum have been investigated in obsessive-compulsive disorder, schizophrenia, major depressive disorder and bipolar disorder, no study has addressed callosal aberrations in all diseases in a single study. Moreover, results from pairwise comparisons (patients vs. controls) show some inconsistencies, possibly related to the parcellation methods to divide the corpus callosum into subregions. The main aim of the present paper was to uncover highly localized callosal characteristics for each condition (i.e. obsessive-compulsive disorder, schizophrenia, major depressive disorder and bipolar disorder) as compared either to healthy control subjects or to each other. For this purpose, we did not rely on any sub-callosal parcellation method, but applied a well-validated approach measuring callosal thickness at 100 equidistant locations along the whole midline of the corpus callosum. One hundred and twenty patients (30 in each disorder) as well as 30 controls were recruited for the study. All groups were closely matched for age and gender, and the analyses were performed controlling for the impact of antipsychotic treatment and illness duration. There was a significant main effect of group along the whole callosal surface. Pairwise *post hoc* comparisons revealed that, compared to controls, patients with obsessive-compulsive disorder had the thinnest corpora callosa with significant effects almost on the entire callosal structure. Patients with schizophrenia also showed thinner corpora callosa than controls but effects were confined to the isthmus and the anterior part of the splenium. No significant differences were found in both major depressive disorder and bipolar disorder patients compared to controls. When comparing the disease groups to each other, the corpus callosum was thinner in obsessive-compulsive disorder patients than in any other group. The effect was evident across the entire corpus callosum, with the exception of the posterior body. Altogether, our study suggests that the corpus callosum is highly changed in obsessive-compulsive disorder, selectively changed in schizophrenia and not changed in bipolar disorder and major depressive disorder. These results shed light on callosal similarities and differences among mental disorders providing valuable insights regarding the involvement of the major brain commissural fibre tract in the pathophysiology of each specific mental illness.

## Teaser

Piras et al. compared highly localized callosal characteristics in four mental disorders (obsessive-compulsive disorder, schizophrenia, bipolar disorder and major depression). They showed that the corpus callosum is highly changed in obsessive-compulsive disorder, selectively changed in schizophrenia and not changed in bipolar disorder and major depression.

## Graphical Abstract

**Graphical Abstract:**
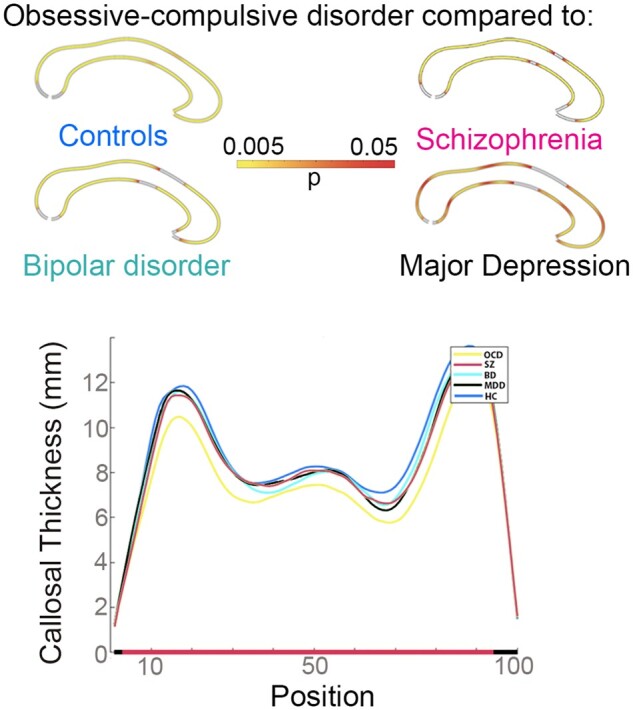

## Introduction

With more than 300 million fibres, the corpus callosum (CC) is the largest commissural white matter fibre tract in the human brain. Ensuing a topographical order, most of its fibres serve homotopic interconnections between the hemispheres^1^^,^^2^ playing a central role in cerebral information transferring and integration. Compartments of the anterior CC, including the rostrum, the genu and the rostral body, connect to prefrontal, premotor, and supplementary motor cortical areas, respectively. Fibres originating from the sensorimotor cortex are assumed to cross the CC through the middle and posterior body, whereas compartments of the posterior CC, including the isthmus and the splenium, connect to temporal, parietal, and occipital cortical regions. However, no macroscopic anatomical landmarks clearly delimit these callosal districts. Thus, several parcellation schemes have been used in neuroimaging studies to partition the CC into anatomically and functionally distinct subareas, in order to achieve more regionally definite measurements. Among them, the widest used method is the well-established Witelson^3^ segmentation criteria, by which the CC is arbitrarily divided into several regions according to maximal length (i.e. in thirds: the anterior third including the genu and the rostrum; the mid-third including the body; the posterior third including the isthmus and the splenium). Others have used a different approach by subjecting midsagittal callosal width measurements, made along the longitudinal axis, to factor analysis for generating a number of smaller clusters.^4^ However, parcellation schemes based on geometrical solutions (e.g. the Witelson scheme) could be biased by local variability in callosal shape,^5^ while measures based on statistically defined internal cohesiveness (using factor analysis) could produce factors that do not necessarily correspond to any functional boundaries. Such variability in anatomical sectioning affects structural MRI studies exploring CC morphology in mental disorders, contributing to inconsistent results. Indeed, despite the amount of interest that CC morphology has reached from the second half of the last century, a general consensus about its involvement in the pathophysiology of psychiatry phenomenology is still lacking.

Mental disorders are characterized, with different extent, by several and often overlapping clinical manifestations.^6^ These includes impairments in complex behaviours (e.g. social interactions and relationships), thought (e.g. reasoning and abstract thinking), high cognitive processes (e.g. attention, working memory, executive functions and inhibitory control), perception (e.g. illusions and hallucinations), mood regulation (e.g. mania or depression) and motor functions (e.g. psychomotor agitation/retardation, catatonia, compulsions). Cortical brain regions, from the most anterior prefrontal cortex to the most posterior occipital areas, are involved in and mediate these processes, requiring a finely coordinated activity for a proper functioning. Providing the physiological substrate for such homotopic inter-hemispheric cortical synchronizations,^7^ CC damage was therefore proposed to be differently involved in the pathophysiology of several mental disorders.

Several pieces of evidence revealed CC microstructural, volumetric and shape alterations in different disorders such as obsessive-compulsive disorder^7–17^ (OCD), schizophrenia^18–28^ (SZ), bipolar disorder^30–39^ (BD) and major depressive disorder^40–47^ (MDD). Specifically, evidence of CC involvement in SZ pathophysiology comes from the very first histological investigations^48^ up to recent studies involving advanced MRI methodologies.^28^ A recent review^28^ and a meta-analysis^49^ suggest that reduced callosal area characterize first episode drug naïve patients diagnosed with SZ. Increasing evidence implicates CC as a key component for the pathophysiology of mood disorders, indicating a disconnection between cortical regions as responsible for impairments in emotional processing.^50^ Specifically, structural MRI studies found reduced CC volume,^33^^,^^51^ signal intensity^31^ and microstructural integrity^36^^,^^39^ (see Bellani et al., 2009 for a Review) in patients suffering from BD, suggesting altered inter-hemispheric connectivity of bilateral homologous cortical areas. In MDD evidence suggests that CC abnormalities are present from the earlier^46^ up to the chronic stages of the illness.^52^ Finally, abnormalities of CC have recently received growing attention in OCD with evidence of either brain microstructural^53^ or morphological abnormalities.^15^

Brain structural^54^ and genetic^55^^,^^56^ studies indicate transdiagnostic overlaps among mental disorders, challenging the view that each brain disorder is exclusively characterized by specific patterns of brain alterations as well as by specific constellations of clinical symptoms.^57–59^ Since the diagnostic process is still based on a descriptive collection of such symptoms to categorize patients, the quantitative measurement of brain neuroanatomical similarities and differences between mental disorders represents a new frontier for psychiatric research. Among CC morphometric studies, the vast majority has been focussed on case–control analyses of one specific mental disorder at a time. As far as we know, to date no study has compared CC morphology among different conditions.

Here, we aimed at uncovering specifically localized CC morphological characteristics for different mental disorders, as compared to healthy control subjects (HC). In order to circumvent the risk of defining callosal sections with controversial fibre distribution, to avoid shape-induced biases, and to increase the spatial resolution of callosal measurements, we did not rely on any parcellation scheme. Indeed, we did perform analyses along the whole midline CC boundaries morphology applying an anatomical mesh-based geometrical modelling method to compute 100 pointwise indicators for callosal thickness.^60^^,^^61^ Furthermore, as no other study did, we compared CC abnormalities^6–54^ across patients diagnosed with OCD, SZ, MDD and BD, controlling for the potential confounding effect of stable antipsychotic drug dosages.^62^

## Materials and methods

We used a priori power calculation in G*power to determine the minimum sample size needed for the study. Results indicated that a total sample size of at least 136 subjects (about 27 participants in each group) and a critical *F*(4; 126) = 1.387 has an actual power greater than 97% power to detect differences among groups, with a two tailed alpha value <0.05.

### Subjects

We recruited 150 subjects (i.e. 120 patients and 30 HC) for the study. Patient groups included 30 participants diagnosed with SZ, 30 diagnosed with BD, 30 diagnosed with MDD and 30 diagnosed with OCD. In all diagnostic groups, individual patients were matched one to one for gender (100% concordance) and age (±1 year). Patients were preliminarily seen in outpatient clinics in central Italy and diagnosed according to the DSM-IV-TR.^63^ Psychiatrists who were treating the patients and knew their clinical history made a preliminary diagnosis of each patient. The clinicians were blind to the aims of the study. All diagnoses were confirmed at the Laboratory of Neuropsychiatry of the IRCCS Santa Lucia Foundation in Rome by a senior clinical psychiatrist (G.S.) using the structured clinical interview for DSM-IV-TR patient edition (SCID-P).^64^ In case of disagreement between the senior psychiatrist and the clinicians who had made the preliminary diagnosis, more data were requested to help resolve the discrepancies and the diagnostic process continued until a final consensus diagnosis was assigned (agreement among raters Cohen’s *k* > 0.80). If the diagnosticians could not reach an agreement, the patient was removed from the sample.

We also recruited 30 HC in the same geographical area, one to one paired-matched with the patients for gender (100% concordance) and age (±1 year). For all participants, inclusion criteria were (i) age between 18 and 65 years; (ii) at least five years of education; and (iii) suitability for MRI scanning. Exclusion criteria were (i) history of alcohol or drug abuse in the two years before the assessment; (ii) lifetime drug dependence; (iii) traumatic head injury with loss of consciousness; (iv) past or present major medical illness or neurological disorders; (v) any (for HC) or additional (for patients) psychiatric disorder or mental retardation; (vi) dementia or cognitive deterioration according to DSM-IV-TR criteria, and Mini-Mental State Examination (MMSE)^65^ score < 25, consistent with normative data in the Italian population^66^; (vii) low quality of T_1_-weighted images (i.e. presence of motion or scanner-generated artefacts); and (viii) any potential brain abnormality or microvascular lesion as apparent on conventional fluid attenuated inversion recovery (FLAIR) scans; in particular, the presence, severity and location of vascular lesions were computed according to the semi-automated method recently published by our group.^67^

The study was approved and undertaken in accordance with the guidance of our local Ethics Committee and written consent was obtained from all participants after a full explanation of the procedures of the study.

### Clinical assessment

Information on clinical history was obtained from the patients, their relatives, psychiatrists and their clinical charts. All HC were screened for a current or lifetime history of DSM-IV-TR Axis I and II disorders using the Structured Clinical Interview for DSM-IV-TR Axis I Disorders, Research Version, Non-patient Edition (SCID-I-NP)^68^ and Structured Clinical Interview for DSM-IV Axis II Personality Disorders (SCID-II)^69^; they were also assessed to confirm that no first-degree relative had a history of OCD, mood- or SZ-related disorders.

For all 150 participants, global cognitive functioning was assessed by the MMSE.^65^ All patients were under stable pharmacologic treatment for at least six months, usually polypharmacy, including treatment with lithium or other mood-stabilizing agents, antidepressants and stable oral dosages of one or more antipsychotics. Illness duration was defined as the age at the MRI scanner time minus age at onset of first symptoms. Symptom severity, chlorpromazine equivalent dosage and illness duration are reported in Table 1.

**Table 1:** Sociodemographic and clinical characteristics of 30 patients diagnosed with OCD, 30 with SZ, 30 with BD, 30 with MDD and 30 HC

	OCD (*N* = 30)	SZ (*N* = 30)	BD (*N* = 30)	MDD (*N* = 30)	HC (*N* = 30)	*x* ^2^, *t* or *F*	Df	*P*
Male, *N* (%)	15 (50%)	15 (50%)	15 (50%)	15 (50%)	15 (50%)	0.00	4	1.000
Age, mean (SD)	39.87 (10.6)	40.27 (9.7)	40.27 (12)	41.02 (10.8)	40.77 (13.9)	0.05	4; 145	0.995
Educational level (years), mean (SD)	13.03 (3.5)	12.43 (3.4)	14.7 (2.7)	13.17 (3.9)	15.97 (3.3)	5.5	4; 145	0.0004
Duration of illness (years), mean (SD)	19.21 (12.3)	24.1 (9)	12 (10)	9.57 (9.7)	–	12.42	3; 114	<0.0001
Clorpromazine Equivalent (mg), mean (SD)	61.1 (105.7)	118.54 (209.4)	80.9 (116.4)	23.21 (53.5)	–	2.38	3; 96	0.074
HAM-D score, mean (SD)	–	–	8.5 (6.2)	16.5 (6.7)	–	−7.98	57	<0.0001
YMRS score, mean (SD)	–	–	3 (2.7)	–	–	–	–	–
Y-BOCS, mean (SD)	27.72 (7.9)	–	–	–	–	–	–	–
PANSS, mean (SD)	–	77.5 (18.2)	–	–	–	–	–	–

df, degrees of freedom; HAM-D, Hamilton Depression Rating Scale; PANSS, Positive and Negative Syndrome Scale; SD, standard deviation; YMRS, Young Mania Rating Scale; Y-BOCS, Yale-Brown Obsessive Compulsive Severity Scale.

### MRI image acquisition and pre-processing

All 150 participants underwent the same imaging protocol, which included 3D T_1_-weighted, T_2_-weighted and FLAIR sequences using a 3T Achieva MR scanner (Philips Medical Systems, Best, The Netherlands) with a 32-channel receiving-only head coil. Whole-brain T1-weighted images were obtained using a fast-field echo sequence (echo time/repetition: time = 5.3/11 ms, flip angle = 9°, voxel size = 1 × 1 × 1 mm^3^). T_2_ and FLAIR sequences were acquired to screen for pathologies that would result in exclusion as described above. The T_1_-weighted images were preprocessed using SPM12 routines (www.fil.ion.ucl.ac.uk/spm/) in order to correct for magnetic field inhomogeneities and to ensure linear spatial alignment to MNI space using six-parameter rigid-body transformations.^70^^,^^71^ In addition, the total intracranial volumes (TIV) were estimated by skull-stripping the images and computing the volume using the FSLstats toolbox (https://fsl.fmrib.ox.ac.uk/fsl/fslwiki/Fslutils#Tools) for later inclusion into the statistical model.

Subsequently, the point-wise callosal thickness was estimated in the preprocessed images as detailed elsewhere.^60^^,^^72^^,^^73^ Briefly, the upper and lower callosal boundaries were manually outlined by two experienced rater (DV and FK) in the midsagittal section of each brain. The intra-rater reliability for these manual traces was high (Cohen’s *k* = 0.90). The resulting upper and lower outlines were then resampled into 100 equidistant surface points each, and a midline was computed as the spatial average of these boundaries. Finally, the distances between 100 corresponding surface points from the upper and lower outline to this new midline were calculated as callosal distance values (CDVs, i.e. a measure of CC thickness) and entered as the dependent variable in the statistical analysis.

### Statistical analysis

Comparisons among groups on sociodemographic variables and clinical characteristics were performed using ANOVA, student’s *t* or chi-square tests, using StatView statistical software (https://statview.software.informer.com), considering *P* < 0.05 as statistical threshold for significance.

Differences in point-wise callosal thickness among all groups were investigated in an ANCOVA using a mass-univariate general linear model. The 100 pointwise CDVs were considered the dependent variable(s) and the diagnosis was the fixed factor. Demographic (i.e. age, educational level), TIV and clinical variables (i.e. duration of illness and chlorpromazine equivalent dosage) were entered as covariates. In order to account for the inter-individual difference in clinical variables among patients (range of continuous values) and HC (only 0), the duration of illness and the chlorpromazine equivalent dosage were standardized by centering patients measures (distances from the mean) to their group mean (considered as 0). By rescaling these variables, we accounted for the inter-individual variability within the patient groups due to differences in illness duration and medication dose, while retaining the overall differences between patients groups and HC. We did not include sex as a covariate since it was 100% matched and highly correlated to TIV (*r*^2^ = 0.42, *P* < 0.0001), as already assessed also by previous studies.^61^^,^^74^

*Post hoc* comparisons were performed in case of significant ANCOVA results, holding the same set of covariates and comparing two groups at time for each CDVs, with univariate general linear models. Specifically, when *post hoc* comparisons included only patient samples (then without HC comparisons), duration of illness and chlorpromazine equivalent dosage were not standardized and considered as raw values.

As statistical tests were made at hundreds of CC surface points and as adjacent data points are highly correlated, statistical results were corrected for multiple comparisons using False Discovery Rate (FDR), with a 5% maximum allowable FDR.^75^^,^^76^

### Data availability

De-identified data are available from the corresponding author upon reasonable request.

## Results

Sociodemographic and clinical characteristics are reported in Table 1. As expected from the matching procedure, the groups did not differ for age and gender, while a significant difference was found for the educational level. Patients’ groups were also different in terms of illness duration and drug treatment (except for mood-stabilizers diverse from lithium), while the difference for chlorpromazine equivalent and benzodiazepine dosages approached statistical significance (Table 1).

**Figure 1:**
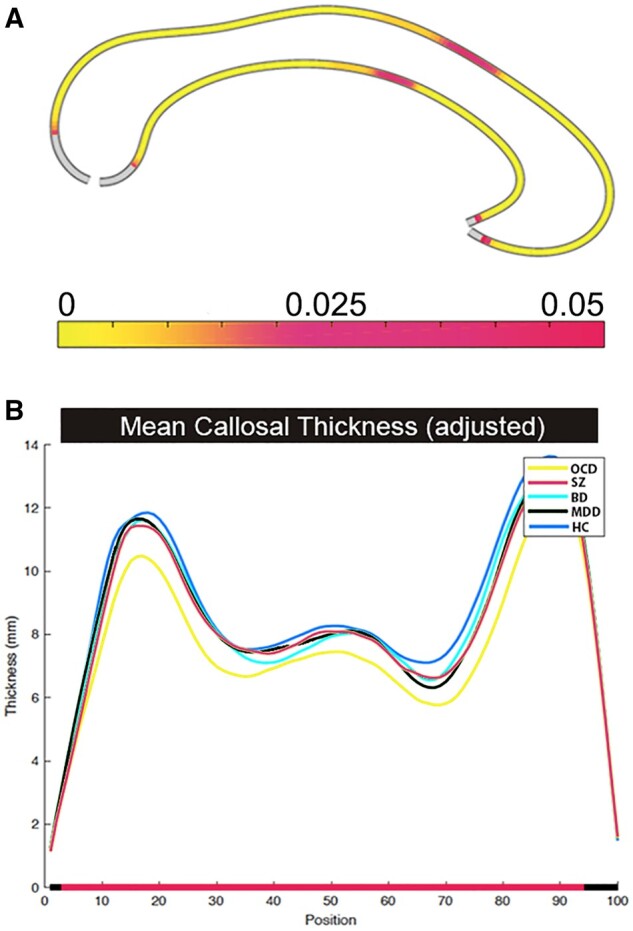
**Differences in mean callosal thickness (adjusted) between HC, SZ, OCD, BD and MDD.** (**A**) Significant group differences after FDR correction for multiple comparisons mapped back on the callosal outlines; The colour bar shows the corrected *P*-values, where magenta represents the weaker and yellow the strongest statistical significance. (**B**) Groups means of the callosal thickness at 100 points from the tip of the rostrum (Position 1) to the bottom of the splenium (Position 100) in healthy control (HC), obsessive-compulsive (OCD), schizophrenic (SZ), unipolar (MDD) and bipolar (BD) mood disorder groups.

Figure 1A shows the results of the ANCOVA. We found highly statistically significant morphological group differences along the vast majority of the CC (*P*_FDRcorr_ < 0.005 for all comparisons), and slightly less significant differences in a little segment along the middle body surfaces (0.005 < *P*_FDRcorr_ < 0.045). The very anterior and posterior ends of the CC did not differ among groups, however, these regions may be less sensitive as they also appear unaffected in severe neurodegenerative conditions.^77^Figure 1B shows mean CDVs for each diagnosis along the callosal axis from the tip of the rostrum to the bottom of the splenium. Overall, the lowest values were evident in OCD, whereas the highest were observed in HC and intermediate values in MDD and BD.

**Figure 2:**
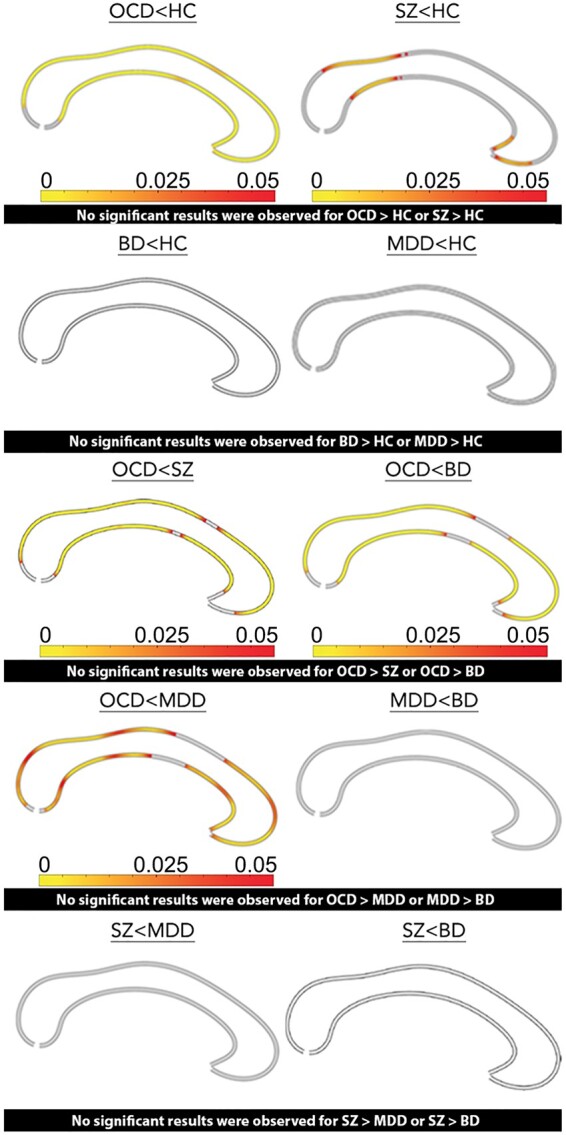
**Pairwise comparisons of callosal thickness between HC, SZ, OCD, BD and MDD.** Significant group differences are colour-coded and mapped onto the callosal outlines for the post hoc group comparisons, performed among of healthy control (HC), obsessive-compulsive (OCD), schizophrenic (SZ), unipolar (MDD) and bipolar (BD) mood disorder; The colour bar shows the FDR-corrected *P*-values, where red represents the weaker and yellow the strongest statistical significance.

*Post hoc* comparisons (Fig. 2) show that OCD and SZ were characterized by significantly thinned CC compared to HC. These effects were evident along the entire structure in OCD (*P*_FDRcorr_ < 0.015 for all comparisons), but loco-regional in SZ [i.e. along the isthmus (0.005 < *P*_FDRcorr_ < 0.035) and the rostrum (0.015 < *P*_FDRcorr_ < 0.03)]. No significant differences were observed when comparing MDD, BD and HC. The comparisons among patient groups indicated that the CC was significantly thinned along the entire structure in OCD, with the exception of the middle body, compared to SZ (*P*_FDRcorr_ < 0.015 for all comparisons), MDD (0.01 < *P*_FDRcorr_ < 0.045) and BD (0.01 < *P*_FDRcorr_ < 0.045) (Fig. 2). The extension of similar morphology of the middle body increased from OCD to SZ to BD to MDD.

## Discussion

Here we explored highly localized CC morphometric features investigating, in a single study, patients diagnosed with OCD, SZ, BD, MDD and HC. We found three main results: first, CC thickness is reduced in both OCD and SZ, but not in BD or MDD, as compared to HC; second, these abnormalities are widespread in OCD and highly localized on the isthmus and rostrum surfaces in SZ; third, OCD have thinned CC along the entire surface except for the middle body, as compared to SZ, BD and MDD. Thus, we observed that CC morphology is highly abnormal in OCD, selectively abnormal in SZ and preserved in BD and MDD.

Although previous studies have investigated callosal size in OCD,^15^ SZ,^21^^,^^22^ BD^30^ and MDD,^41^ to the best of our knowledge, our study is the first to compare callosal thickness measures across all these major mental disorders.

Using different techniques, CC macrostructural^15^^,^^78^ alterations have been already described in OCD compared to HC, on the splenium, the isthmus and the anterior body and related to impaired inter-hemispheric connections among occipital, parieto-temporal and orbito-frontal associative cortices.^1^^,^^2^^,^^79^ Posterior CC changes in OCD have been related to impairments in visual-spatial performances^15^ while anterior CC abnormalities have been linked to executive prefrontal lobe disfunctions.^80^ Indeed, executive abilities such as response inhibition and set shifting, as well as working memory and planning, are often reported impaired in adults with OCD.^81–84^ Our results shed light on the potential involvement of the anterior CC in the pathophysiological processes mediating these cognitive disfunctions. Apart from the impairment of inter-hemispheric connections among cerebral associative cortices in OCD, here we describe an additional change of sensorimotor cortices connections, as mediated by CC fibres crossing the middle body. Sensorimotor cortices are part of the cortico-striato-thalamo-cortical loop, that has been widely described as a critical network in OCD pathophysiology.^85^^,^^86^ Indeed, reduction of sensorimotor cortical inhibition upon cortico-striato-thalamo-cortical impairs the ability to suppress internally triggered intrusive and repetitive movements (compulsion) and thoughts (obsessions).^87^ Thus, impairments in inter-hemispheric sensorimotor communication and integration may be also involved in the cortical inhibition impairment present in OCD.

In patients diagnosed with SZ^21^^,^^22^ in comparison with HC, CC structural changes have been previously described for the most anterior and for posterior portions of the CC. Here, we strengthen this evidence, confirming the presence of focal white matter changes in SZ.^88^ In particular, differently from OCD, callosal changes found in SZ were highly localized and restricted to the inter-hemispheric connections crossing the CC rostrum and the splenium. Fibres crossing through the rostrum mediate inter-hemispheric transfer of the ventromedial prefrontal and orbitofrontal cortices. Both these cortical regions^89^^,^^90^ are critical for a number of high cognitive processes, such as the representation of reward and value-based decision-making, emotion regulation and for multiple aspects of social cognition (i.e. facial emotion recognition, theory of mind ability and processing of self-relevant information). Such processes have been proved to be dysfunctional in SZ, both in neuroimaging^91^ and neuropsychological studies,^92–95^ and abnormal inter-hemispheric connections among the rostrum may be involved in such impaired functioning. Fibres crossing the isthmus mediate inter-hemispheric connections among posterior parietal cortices (PPC), that are multimodal associative areas also associated to high cognitive processes.^96^ Specifically, PPC functions mediate stimuli representations and the maintenance of attention over stimuli represented in sensory cortices.^97^ Moreover, there is strong evidence that PPC-based functions are central to working memory storage.^98–101^ Both attentional and working memory functions have been widely reported as impaired in SZ,^102^ and our results of thinned CC isthmus in SZ indicate that altered inter-hemispheric PPC connection might be one of the cerebral substrates of such cognitive impairment.

Here we did not find significant CC changes in BD as compared to HC. Previous studies, investigating morphometric features in BD, described alterations in CC thickness,^33^^,^^103–105^ microstructure^106–108^ or interhemispheric connectivity,^109^^,^^110^ though with some inconsistencies.^111^ Moreover, studies focussed on younger^112^ or fist-episode^113^ patients diagnosed with BD did not describe CC size changes but only curvature alterations. In order to explain inconsistences in the results, different sources of heterogeneity should be considered for MRI investigation in BD. Indeed, BD encompasses a broad of symptomatology and disease subtypes, leading to several issues in patient categorization. In particular, some of the citated studies were focussed only on patients diagnosed with BD type I,^32^^,^^103^^,^^104^ while the majority of them completely lacks to specify diagnostic subtype information.^34^^,^^105^^,^^108^^,^^112^^,^^113^ Furthermore, all but one^104^ among the cited studies lack to control statistical analysis for the potential impact of psychiatric/antipsychotic treatment, which effect on white matter structure is well known.^114^ Lastly, differences related to the technique and the measures employed for MRI investigations add a considerable amount of heterogeneity among CC studies, leading to problems in result comparisons. Indeed, the cited morphometric works often relayed on discrete CC parcellation schemes^32^ or used a smaller number of points on the CC surface in order to obtain thickness measurements (i.e. 39 points).^103^^,^^113^ Conversely, we obtained a continuous-like measure of the distances between upper and lower callosal surfaces employing 100 points of measurement, then performing a higher localized analysis along the whole CC surface.

Morphometric studies investigating callosal size in MDD also show a considerable amount of inconsistencies.^40^^,^^41^ Here, we are in line with results of unchanged CC thickness in this mood disorder. Specifically, abnormalities in MDD were found when the patient’s sample was stratified for illness onset^41^ (i.e. early versus late onset) or for familiar form of the diseases.^38^ It could be argued that callosal changes in MDD are present only in specific, or even in the more severe, forms of the disease, being not detectable for all types of MDD.

The third intriguing finding in our study was the significant thinned CC found in OCD patients compared to SZ, BD and MDD, on the majority of the investigated surfaces, indicating extensive CC implication in OCD mechanism in comparison with other major mental disorders. Indeed, only surfaces belonging to the middle body were not different among OCD and SZ, BD or MDD. The widespread changes in CC morphology of patients diagnosed with OCD indicates that interhemispheric integration of both associative and primary sensory visual cortices in OCD was the most impaired among the investigated disorders. This evidence could be useful also from a diagnostic point of view. Indeed, while mental diseases could overlap from a symptomatologic dimensional perspective, they result as categorially different referring to CC morphometric characteristics. Thus, a widespread thinned CC it could reasonably guide the diagnostic process towards a potential diagnosis of OCD.

Referring to callosal similarities among patient samples, fibres connecting pre- and post-central sensorimotor cortices by the middle body^1^^,^^2^^,^^79^ showed more homogeneous measures among patients. Focussed on OCD sample, it should be noted that the morphometric features of this CC area were the least affected as compared to HC. Being abnormally reduced in OCD patients only, it could be argued that inter-hemispheric sensorimotor integration is impaired in OCD but the extent of such impairment is not great enough to be significant in the comparisons with the other mental disorders. Future connectivity investigations should be performed in order to clarify this issue.

### Limitations

Three main issues have to be considered before concluding. First, we didn’t control the analysis for the impact of antidepressant and mood stabiliser medicaments. Although this kind of treatment have been described as mostly related to changes in neuropils and grey matter structures,^115–117^ there is evidence that they may impact white matter as well (e.g. van Velzen et al.^118^). Future studies specifically focussed on the effect of such medications and with an adequate sample size are needed to address this complex issue in further detail. Second, the mental disorders here investigated may be better characterized by their clinical subtypes, in terms of symptom domains or severity, than by main diagnoses. However, stratifying patients in clinical sub-groups, characterized by more homogenous clinical manifestation, requires higher sample size to avoid false negative results. Further studies with larger sample sizes are needed to investigate this issue.

Third, our OCD sample was predominantly composed of patients with a late onset of the disease, according to the cut-off age of 19 proposed by Anholt and colleagues.^119^ Therefore, it could be argued that our results cannot not be generalized to OCD as whole, but limited to the late onset subtype. However, while it has been suggested that early and late onset adult OCD may differ in terms of neuropsychological features and grey matter volume (e.g. Kim et al.^120^), there is no clear evidence that they also differ in terms of white matter structure.

## Conclusion

The comparison of CC morphometric features among OCD, SZ, BD, MDD and HC samples using an ultra-sensitive and highly localized morphometric technique allowed us to finely characterize CC morphometric features in four of the major mental disorders. Our approach revealed for the first-time similarities and differences among these patients, and points towards a thinned CC as a potential biomarker for OCD, as compared either to HC or to other major disorders. These results provide new insight into the involvement of the major brain commissural fibre tract into the pathophysiology of each of these specific mental disorders.

## Funding

The study was partially funded by the Italian Ministry of Health, grants Ricerca Corrente, year 2021.

## Competing interests

All authors declare no competing interests.

## Abbreviations

BD =: bipolar disorder
CC =: corpus callosum
CDV =: callosal distance values
CSTC =: cortico-striato-thalamo-cortical
FDR =: false discovery rate
FLAIR =: fluid attenuated inversion recovery
HC =: healthy controls
MDD =: major depressive disorder
MMSE =: mini-mental state examination
OCD =: obsessive-compulsive disorder
PPC =: posterior parietal cortices
SZ =: schizophrenia
TIV =: total intracranial volumes
